# Sir John Williams at Cardiff

**Published:** 1900-10-20

**Authors:** 


### The Hospital

A Journal of

The Medical Sciences and Hospital Administration.

Vol. XXIX.—No. 734.] Edited by Sir Henry Burdett, K.C.B., and Solomon C. Smith, M.D., M.R.C.P. [October 20, 1900.

### Sir John Williams at Cardiff.

The introductory addresses delivered to medical students at the opening of the session just commenced have, as a rule, been very much of what may be called, without undue profaneness, the standard quality; and would have justified many of the hearers in such an appreciation of them as that of the Northern Farmer:—

"An' I thowt a said whot a owt to 'a said an' I coom'd awaay."

Audiences have been duly told of the value of diligence, of the importance of time, of the weight of medical responsibilities, of the necessity of due preparation for their discharge, of the essential characteristics of a scientific training, and so on; with perhaps an occasional *excursus* upon the rewards which observation of the speaker's precepts might be expected to bring in its train, or upon the consolations likely to be afforded by the performance of duty, even in cases where the rewards in question tarried unexpectedly upon their way. All this we have heard before. Much of it is true; some of it is, perhaps, obvious; some of it, there is reason to fear, may be altogether fallacious, and must ever remain so in a world where "time and chance happeneth unto all." It was reserved for Sir John Williams, in his introductory Address delivered at the University College of South Wales and Monmouthshire, to strike a somewhat new vein—and if he did not altogether escape from the accustomed platitudes, at least to reserve them as the copingstones of a structure of a very different order—in which he dwelt upon educational topics of real and varied interest. He commenced with an outburst of just and righteous indignation against the newspaper press, for its sordid complicity in the frauds of advertising quacks, concerning which he used language of plain truth, which cannot be too widely circulated or too attentively considered. He pointed out that nearly all newspapers, religious or secular, except a few of the latter, constantly contain accounts of impossible cures, and statements of effects produced by drugs which everybody possessed of the most elementary knowledge of the laws of health would know to be entirely devoid of foundation. These statements are published in newspapers which profess to teach, and to lead and form public opinion, which are the property of men who hold more or less influential positions, and are edited, some by clergymen, some by ministers, some by laymen. The statements referred to are sanctioned by silence, in speech, or in writing, by everyone concerned in the publications in which they appear. "When I find," continued Sir John, "the religious newspapers of the Principality besmeared with such advertisements, some of them not only false, but foul and wicked, I cannot help feeling the blush of shame as I bear in mind under whose ægis they are published." The "religion" of "religious" newspapers belongs, we fear, chiefly to the creed which may be comprehensively described as "Krugerism," in which the only end of godliness is conceived to be the getting of gain. Its followers are probably impervious to shame; but it is at least worth trying whether they cannot be discredited among their dupes; and any effort in this direction, made in the Principality by a Welshman so distinguished as Sir John Williams, deserves to be hailed with gratitude by all who hold truth and honesty in esteem.

From this unsavoury but wholesome topic, Sir John passed by an easy transition to the consideration of educational questions; and after a description of his own school days, such that it would make all who read it long to be boys again, if only they could be placed in equally favourable circumstances, he discussed at some length the value of languages as means of mental training. Greek and Latin he holds to be of small value as parts of medical education; and he quoted an unnamed "distinguished writer and scholar who has recently passed away" in support of this contention, which, we venture to think, is more plausible than accurate. He expressed a well-founded belief that the "power of reading" both French and German might be acquired in the time now commonly devoted to Latin and Greek; and his quoted authority maintained that "in order to speak English with accuracy and precision we have but one rule to follow—to pay strict attention to usage." We cannot but inquire whose usage? The value of Latin never depended so much upon anything in the language itself as upon the fact that, in former times, it was taught thoroughly to those to whom it was taught at all. The learners were made to acquire precise notions of the force, meanings, and values of words. For this purpose, one language is as good as another; but no one who does not thoroughly understand at least one language can ever thoroughly understand anything else. Words are the representatives of ideas; and, if the words are of uncertain value, the ideas themselves must be vague and illusory. Perhaps Sir John's "scholar" lived in a scholastic atmosphere in which usage may have been based upon learning. What is it in ordinary life? What does an average medical student mean by a "lesion"? If Sir John were to turn from the advertisements which he condemns to the news columns of the same papers he would find that every vulgar crime was a "mystery"; that every accident was "appalling"; that every unusual occurrence was "phenomenal"; that every error was a "fallacy"; that every hypothesis was a "theory," and so on. The paragraph writers are fertile and ingenious, but they have never learnt a language, and they do not know the meanings of common words. Sir John recommends the study of Welsh, and, we are glad to see, recommends that it should be studied in such a way that it would fulfil all the educational requirements of the case.

